# Supplementary material for: Psychological resilience mediates the association between sleep quality and anxiety symptoms: A repeated measures study in college students
Source: PLoS One. 2023 Oct 25;18(10):e0292887. doi: 10.1371/journal.pone.0292887 (PMC10599530; doi:10.1371/journal.pone.0292887)
Supplement: Attachment — Submitted filename: Plos one.docx [file pone.0292887.s002.docx]

Dear Editor,

Many thanks for the opportunity to review this paper. The authors investigated psychological resilience mediates the association between sleep quality and anxiety symptoms. The paper is in the interest of the journal. Please see my comments below:

*‘This study complied with the Declaration of Helsinki and approved by the 76 Ethics Committee of Fujian medical university.’*

- Reference must be added
- Line 154 shows a letter error. (PIQI)
- They stated that the scales were applied repeatedly. It has been reported that there is a significant difference between the two measurements in GAD scores. It is recommended that this measurement difference be discussed.

*‘Another cross-sectional study also found a significant association between PSQI and GAD scores’*

- The direction of the relationship in the statement should be explained.

There are publications in the literature suggesting that sleep quality has a mediating role in anxiety symptoms. It should be explained why the mediation model was planned on the path from stressful life events to anxiety symptoms. The chronological emergence of the factors included in the mediation model should be mentioned. Chronologically, using the previously revealed factor as a mediator does not seem to be very compatible with the model.

*'A potential mechanism by which poor sleep quality affects mental health is that poor sleep quality may affect the neuroendocrine system, particularly leading to activation of the hypothalamic-pituitary-adrenal axis and elevated glucocorticoids, thereby affecting mental health.'*

- This situation is not included in the study data or the introduction part of the article. It is recommended to explain the result for another possible reason or to make a connection with the subject in the introduction.

*‘Studies also 200 have found a negative correlation between psychological resilience and its various dimensions and anxiety. The possible reason for this is that psychological resilience contributes to positive emotional recovery’*

- This statement should be detailed. Positive emotional recovery is often used for depressive processes. Its connection with anxiety should be detailed.

*‘Further, some other studies have found an association between sleep quality and psychological resilience. The possible reason for this is that poor sleep quality leads to poor psychological stress capacity.’*

- In this study, in which cross-sectional data were analyzed, it is not a direct result that low sleep quality causes psychological stress. However, it can be said that they are related. The explanation should be made with the support of the literature.

*‘This study highlights the importance of improving sleep quality while enhancing psychological resilience to prevent the onset of anxiety symptoms in college students.’*

- From what data is the conclusion about the onset of anxiety symptoms derived? The study's statistical analysis is cross-sectional and it doesn't seem easy to predict the cause-effect relationship.

,

*'Therefore, there is a need for prospective studies to explore the association between sleep quality and anxiety.'*

- In the study presented in the longitudinal design, but there is only one measurement statistical analysis. It is recommended to elucidate the contribution of the longitudinal pattern to the study.

All in all, the authors could improve the manuscript after appropriate revisions.

Best regards
