# Supplementary material for: Psychological resilience mediates the association between sleep quality and anxiety symptoms: A repeated measures study in college students
Source: PLoS One. 2023 Oct 25;18(10):e0292887. doi: 10.1371/journal.pone.0292887 (PMC10599530; doi:10.1371/journal.pone.0292887)
Supplement: Attachment — Submitted filename: Plos one.docx [file pone.0292887.s004.docx]

Dear Editor, my comments on the reviewer responses are below:

- In the study presented in the longitudinal design, there is only one measurement statistical analysis. It is recommended to elucidate the contribution of the longitudinal pattern to the study. Authors' explanation is that 'sleep quality, resilience and anxiety symptoms were all measured twice. We based on this to explore the dynamic association between sleep quality and anxiety symptoms among college students.' The statistical analysis should be discussed for other measurements.
- The chronological emergence of the factors included in the mediation model should be mentioned. Chronologically, using the previously revealed factor as a mediator does not seem compatible with the model. The authors' explanation is that 'In this study, what we mainly explored is that psychological resilience played the mediating role in this association between sleep quality and anxiety symptoms. Several studies have examined the mediating role played by psychological resilience in the association between external influences and mental health. Based on that, we explored the association of sleep quality with anxiety symptoms and the mediating role of psychological resilience in this association.' Taking references from previous studies is partially accepted. However, its compliance with the basic principles of mediation analysis should be explained within the framework of statistical science.
- In the study, although the participants were observed for a certain period, cross-sectional data was analyzed. Therefore, it does not seem compatible with the longitudinal pattern of the study. The emphasis on the longitudinal pattern should be changed in the title and content of the study.
